# Analytical solution of overlying pipe deformation caused by tunnel excavation based on Pasternak foundation model

**DOI:** 10.1038/s41598-022-26635-8

**Published:** 2023-01-17

**Authors:** Daxi Fu, Bo Deng, Minghui Yang, Binbin Zhen

**Affiliations:** 1 School of Civil Engineering of Beijing Jiaotong University, Beijing, 100044 People’s Republic of China; 2 Henan Provincial Transportation Planning and Design Institute Co., Ltd, Zhengzhou, 450015 People’s Republic of China; 3 College of Civil Engineering, University of South China, Hengyang, 421001 Hunan People’s Republic of China; 4 College of Civil Engineering, Hunan University, Changsha, 410082 People’s Republic of China

**Keywords:** Civil engineering, Engineering

## Abstract

The existing tunnel construction causes stratum deformation, which in turn leads to additional deformation and internal force of the overlying pipeline, thus increasing the risk of pipeline accidents. Then, how to correctly calculate the deformation and internal force of pipeline is the key to pipeline safety evaluation. To this end, this study firstly used the Pasternak foundation beam model to simulate the interaction of pipeline and soil, and divided the pipeline into the void area (i.e., pipeline-soil detachment) and the coordination area (i.e., pipeline-soil is always deformed together) between pipeline and soil. The differential equation of pipeline deflection for the void area and the coordination area were established respectively, and the solutions of pipeline deflection, the internal force of pipeline and the width of pipeline-soil void area were presented. Subsequently, the accuracy of the proposed method was verified by comparing with the available model and field test data, and it is found that the calculation results are too conservative without considering the pipeline-soil voiding phenomenon. Finally, the detailed parametric analysis was conducted. The results show that the pipeline deflection decreases with the increase of the pipeline-tunnel spacing between pipeline and tunnel, the pipeline bending stiffness and the soil elastic modulus, but increases with the increase of the formation loss rate, and the width of pipeline-soil void area increases with the increase of the pipeline-tunnel spacing, the pipeline bending stiffness and the soil elastic modulus.

## Introduction

Tunnel excavation will lead to the redistribution of stress in surrounding soil, break the initial stress balance, make the surrounding soil slip or displacement, and then drive the surrounding underground pipeline deformation, and in serious cases lead to pipeline damage and fracture. In recent decades, with the development of tunnel construction in many countries throughout the world, engineering accidents caused by underground pipeline damage caused by tunnel construction have been reported frequently^1–4^. To deeply analyze the influence of tunnel construction on adjacent existing pipelines, and put forward a more reliable prediction method to provide a strong basis for the design and decision of the actual project, it is of great significance to carry out a more comprehensive and systematic study on related issues.

At present, many scholars at home and abroad have noticed the deformation of existing overburden pipelines caused by tunnel excavation in underground engineering construction, and have carried out detailed research on this problem, and the main methods used include model test^5–8^, numerical simulation^9–11^ and theoretical analysis^12–20^. By contrast, the theoretical analysis method can effectively provide guiding opinions for engineering because of its clear physical concept. In recent years, in terms of theoretical research, Attewell et al.^12^ first proposed to regard the pipeline as a Winkler foundation beam model, and established the deformation differential control equation by assuming that the joint deformation of the pipe and soil and the free settlement of the stratum obey the Gaussian distribution, and gave the analytical solution of pipe deformation; Liang et al.^13^ established the transverse vibration equation of the pipeline using the two-parameter Pasternak foundation model, and discussed the influence of the foundation shear stiffness on the stability of the pipeline system; Zhang et al.^14^ used the continuous elastic analysis method to simulate the stress and displacement response of the continuous pipeline with joints caused by tunnel excavation in multi-layer soil; Klar et al.^15,16^ considered the nonlinear characteristics of soil stiffness weakening effect and pipeline-soil interaction caused by tunnel excavation, and deduced the expressions of pipeline deformation and bending moment by using elastic continuity theory; Shi et al.^17^ studied the impact of explosion load of adjacent tunnel construction on the safety of pipelines, and based on the dynamic and static analysis of pipelines, established a method to determine the control standard of peak vibration velocity of buried pipelines under explosion load; Zhao et al.^18^ derived a finite difference model for the skewed pipeline deformation, which can consider the influence of shield shell friction and ground loss.

However, most of the above analytical models adopt the elastic foundation beam theory, which assumes that the pipeline is always in close contact with the soil, and the deformation of the pipeline and the soil is coordinated. In fact, due to the deformation difference between pipeline and soil, the soil in a certain area is separated from the bottom of the pipeline, and the stress concentration appears in the critical section of the pipeline and soil, which will further aggravate the volume loss of the soil around the pipeline and increase the deformation of the pipeline, thus threatening the safety of the pipeline^19–21^. Additionally, several test results show that there is a situation of pipeline-soil voiding^22–25^. Vorster^22^ analyzed the influence of sand tunnel excavation on the deformation of the pipeline through the centrifugal test, showing that there is a void phenomenon at the bottom of the pipeline. Marshall^23^ explored the volume loss law of sand tunnels under different stiffness pipes by centrifuge test and elaborated the mechanism of pipeline-soil interaction in the process of tunnel volume loss, and also found that gaps were formed beneath the pipeline. Cheng et al.^25^ carried out centrifugal model test to observe the pipeline-soil void behavior, confirmed the existence of pipeline-soil void phenomenon, and expounded that it is necessary to consider pipeline-soil void in the calculation of pipeline deformation. Unfortunately, only few literatures have incorporated the effect of the pipeline-soil void in the existing analytical solutions, such as Lin et al.^26^ regarded pipeline and soil as Euler–Bernoulli beam and Pasternak foundation respectively, and pipeline-soil void area as another element, the finite difference method was used to solve the pipeline deformation considering the formation of gap and pipeline orientation under the condition of force balance and deformation coordination simultaneously.

Although the above mentioned research is available, the problem of pipeline deformation caused by tunnel excavation has not been well addressed. In view of this, this study expands on previous research by introducing the Pasternak foundation model and semi-infinite beam theory, and establishes the pipeline-soil interaction model with the void formation. Subsequently, based on the critical condition of the void area and the coordination area, the analytical solutions of the pipeline deformation caused by the tunnel excavation and the range of the pipeline-soil void area are put forward, and the calculation results are compared with the existing test results for verifying the proposed method. Finally, the influence of the main factors such as pipeline bending stiffness, soil elastic modulus, pipeline-tunnel spacing and formation loss rate on the pipeline deformation and the expansion of the void area is discussed for reference in engineering design.

## Deformation mechanism of pipeline caused by tunnel underpass

**Figure 1:**
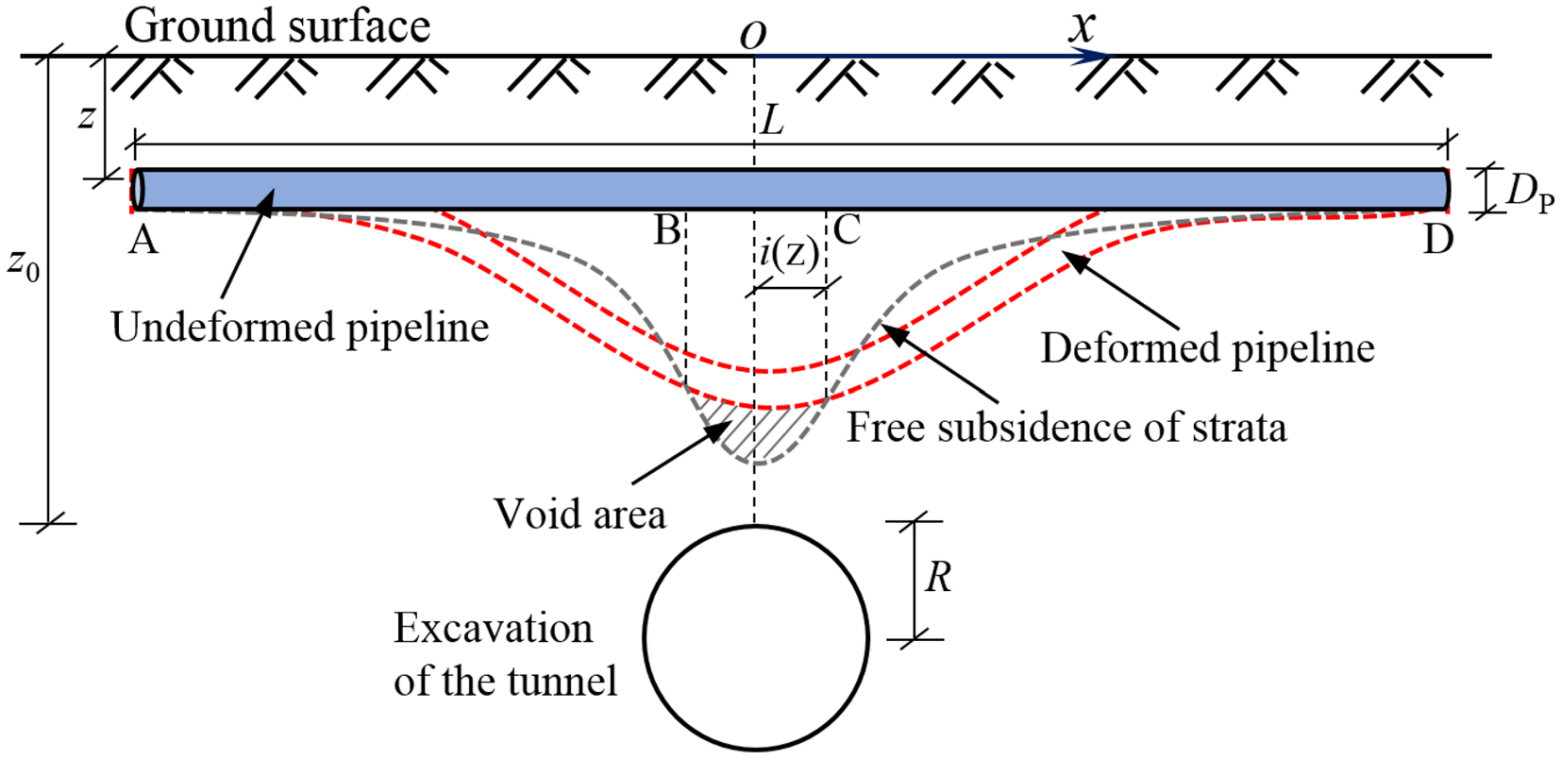
Mechanical model of pipe-soil interaction based on detachment phenomenon.

When the tunnel passes through the existing pipeline (such as natural gas pipe, municipal pipe, etc.), the overlying soil and the pipeline have a certain amount of deformation, and the relative deformation between the pipeline and the tunnel is closely related to the pipeline stiffness. If the pipe stiffness is much greater than the soil stiffness (i.e., the absolute rigid pipe), the surrounding soil deformation is inconsistent with the pipe deformation, resulting in the detachment of pipe and soil. On the contrary, if the stiffness of the pipe is close to the soil (i.e., the absolute flexible pipe), the deformation of the pipe is completely consistent with the deformation of the surrounding soil, and the pipe and soil are always in contact. According to the centrifuge test results of Vorster et al.^22^, the actual pipe-soil contact situation is between the above two. Thus it can be seen that the pipe-soil system can be divided into two areas, namely, the void area from the pipe axis to a certain range outside and the coordination zone (coordinated deformation) outside the area, as shown in Fig. 1.

## Tunnelling-induced pipeline deformation

The tunnel excavation passes through the existing pipeline, which involves the complex tunneling-pipe-soil interaction, so it is difficult to model and solve directly in theory, but the two-stage method can be used to analyze^27^. In the first stage, the vertical displacement of soil caused by tunnel excavation at the pipeline axis is calculated (ignoring the influence of pipeline); In the second stage, the pipe-soil interaction model is established, the soil deformation caused by the first stage is regarded as the external load, and the obtained free displacement of the soil is applied to the pipeline, the vertical load-bearing deformation balance differential equation of the pipeline is established and solved, and then the vertical displacement of the pipeline is obtained. For the convenience of calculation, the following assumptions are made in this study:

1. Only the situation where the pipeline and the tunnel are perpendicular to each other is considered. Since the lateral soil action of the pipeline is small enough to be ignored compared with the vertical action, so it is regarded as a plane strain problem;
2. Both the pipeline and the soil are continuous homogeneous bodies, and the section size change caused by the pipeline deformation is ignored;
3. The pipeline is only affected by the displacement load of the stratum and the gravity of the overlying soil, and the influence of other loads during construction is ignored.

### Elastic foundation model

In the previous two-stage method for the effect of tunnel excavation on the deformation of the overlying pipeline, a reasonable foundation beam model was used in the second stage to simulate the interaction between the pipeline and the soil. Among them, the Winkler elastic foundation beam model is widely used because of its simplicity and clear physical meaning, but this model also has obvious defects, that is, it does not consider the continuity of the foundation soil deformation, and ignores the influence of shear force transfer in the soil on the calculation results, resulting in a certain error between the calculated value and the measured result.

**Figure 2:**
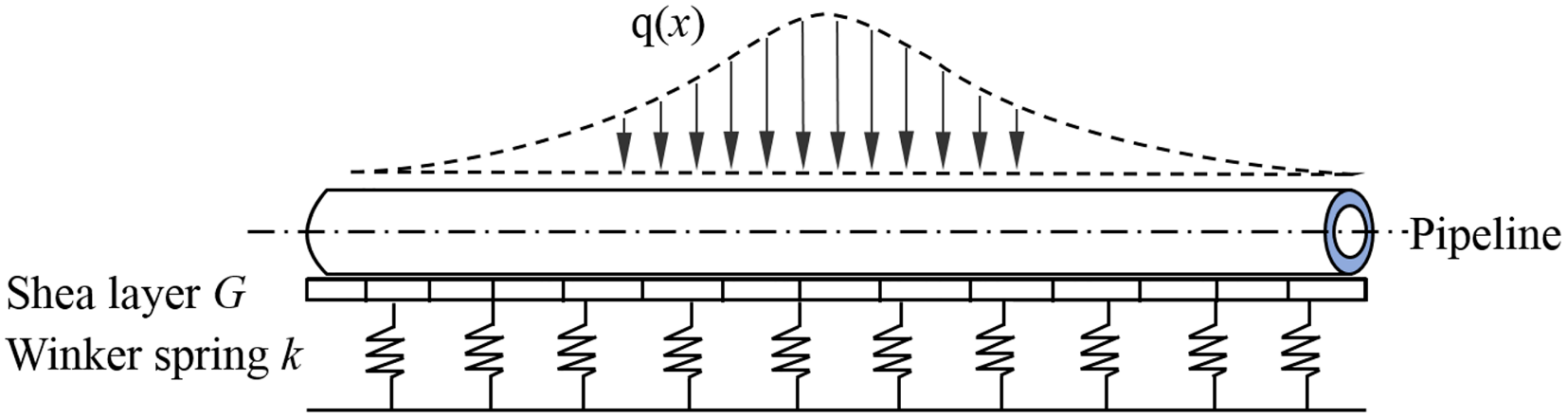
Pasternak foundation model.

**Figure 3:**
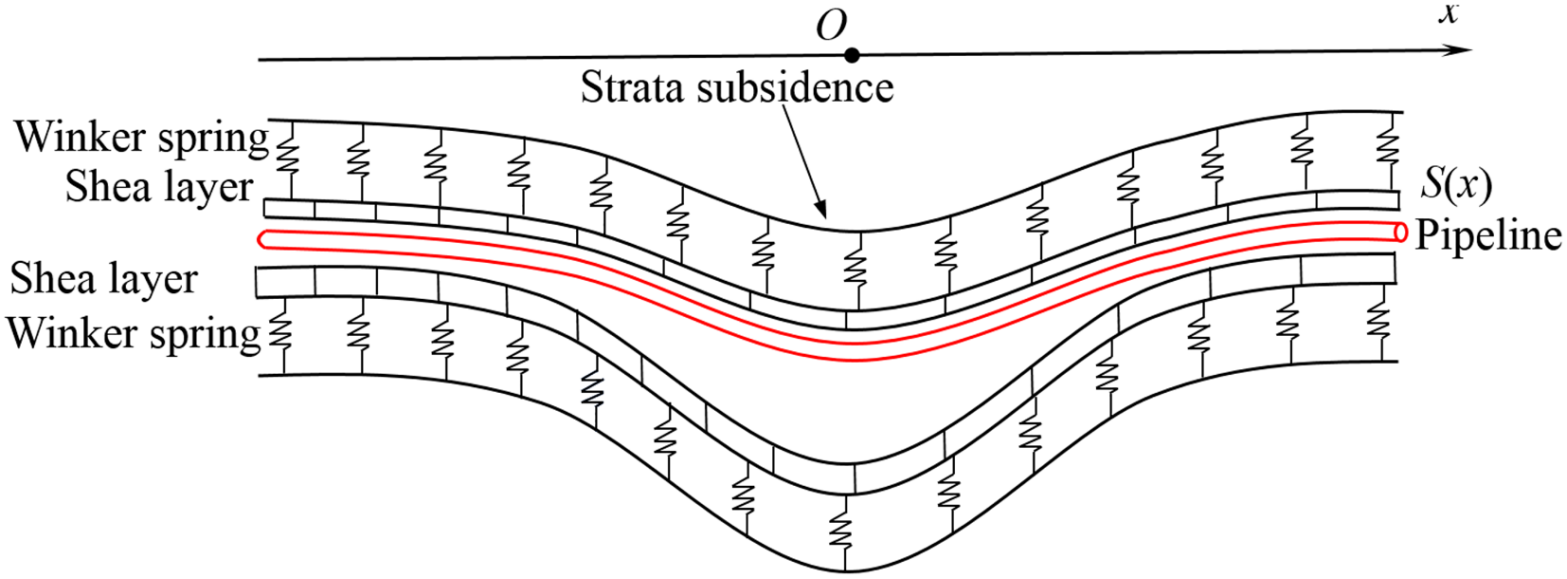
Pipeline-soil interaction model.

Comparing with the Winkler foundation model, the Pasternak foundation model connects the spring elements in the Winkler foundation through a shear layer that only produces transverse shear deformation, and considers the shear interaction between independent springs (see Fig. 2). The Pasternak foundation model is widely used to study soil-structure interaction because it considers the continuity of soil deformation and is more in line with the actual engineering situation. Therefore, this model is also used to simulate the pipeline-soil interaction in this study. As shown in Fig. 3, converting the soil displacement equivalently into the additional load acting on the pipeline, Pasternak^28^ gave the expression of *q*(*x*) as

1 $$ q(x) = - G\frac{{d^{2} S(x)}}{{dx^{2} }} + kS(x) $$

where *x* = lateral horizontal distance from the tunnel axis; *q*(*x*) = distributed load acting on the pipeline at coordinate *x*, which is caused by strata subsidence; *G* = Pasternak foundation reaction coefficient; *S*(*x*) = ground settlement at coordinate *x*; *k* = coefficient of subgrade reaction.

### Analysis model of pipeline deformation

According to the above ideas, the vertical free displacement of soil caused by tunnel excavation can be calculated first. Based on a large number of engineering examples, Peck^29^ found that the ground settlement perpendicular to the tunnel axis direction caused by stratum loss during tunnel excavation roughly conforms to the Gaussian curve distribution

2 $$ S_{0} (x) = \frac{{\pi R^{2} \varepsilon }}{{\sqrt {2\pi } i_{0} }}e^{{\frac{{ - x^{2} }}{{2i_{0}^{2} }}}} $$

where *S*_0_(*x*) = ground surface settlement at coordinate *x*; *R* = tunnel radius; *ε* = formation loss rate caused by tunnel excavation; *i*_0_ = width coefficient of surface settlement trough.

Considering the pipeline is buried at the underground depth *z*, the depth correction is required due to the width of settlement trough is different from that at the surface^30^, so the above Eq. (2) can be modified as

3 $$ i(z) = i_{0} \left( {1 - z/z_{0} } \right)^{n} $$

where *z*_0_, z = buried depth of tunnel and pipeline respectively; *i*(*z*) = width of surface settlement trough at depth *z*; *n* = influence coefficient related to tunnel radius and soil conditions, which typically falls in the range of 0.35–0.85 and 0.85–1.0 for cohesive soil and sandy soil, respectively.

The vertical free displacement of soil at the point of pipeline axis (*x*, *z*) is calculated by coupling Eqs. (1) and (2) as

4 $$ S(x) = \frac{{\pi R^{2} \varepsilon }}{{\sqrt {2\pi } i(z)}}e^{{\frac{{ - x^{2} }}{{2i^{2} (z)}}}} $$

Let $S_{\max } (z) = \frac{{\pi R^{2} \varepsilon }}{{\sqrt {2\pi } i(z)}}$, wherein $S_{\max } (z)$ = maximum ground settlement above the tunnel axis at depth *z*.

For the calculation of pipeline deformation caused by vertical displacement of the ground, the key point is to simulate the interaction between a buried pipeline and the surrounding soil. Currently, the commonly used method is to treat the pipeline as an infinite beam placed on the elastic foundation, and assume that the pipeline is an Euler–Bernoulli beam on the Pasternak elastic foundation. Considering geometric and loading symmetry, just the right-half of the infinite beam (see Fig. 1) is selected for analysis, and the differential control equation of vertical deflection of pipeline should be expressed as^31^

5 $$ E_{{\text{p}}} I_{{\text{p}}} \frac{{d^{4} w(x)}}{{dx^{2} }} + kD_{{\text{P}}} w(x) - GD_{{\text{P}}} \frac{{d^{2} w(c)}}{{dx^{2} }} = q(x)D_{{\text{P}}} $$

where *w*(*x*) = vertical deflection of pipeline at coordinate *x*; *D*_P_ = outer diameter of the pipeline; *E*_p_ = elastic modulus of pipeline; *I*_p_ = section moment of inertia; *G* = Pasternak foundation shear stiffness.

When analyzing the pipeline-soil interaction, Yu et al.^32^ introduced the modified foundation reaction coefficient *k* to consider the buried depth effect as

6 $$ k = \frac{{3.08E_{{\text{s}}} }}{{\eta D_{{\text{p}}} \left( {1 - v_{{\text{s}}}^{2} } \right)}}\left( {\frac{{E_{{\text{s}}} D_{{\text{p}}}^{4} }}{{E_{{\text{p}}} I_{{\text{p}}} }}} \right)^{\frac{1}{8}} $$

where *η* = depth correction factor calculated by $$\eta = \left\{ {\begin{array}{*{20}l} {2.18} \hfill & {z/D_{{\text{p}}} \le 0.5} \hfill \\ {1 + \frac{1}{{1.7z/D_{{\text{p}}} }}} \hfill & {z/D_{{\text{p}}} > 0.5} \hfill \\ \end{array} } \right.$$; *E*_s_, *v*_s_ = elastic modulus and Poisson's ratio of soil respectively.

Tanahashi et al.^33^ suggested the Pasternak foundation shear stiffness *G* for solving the static problem of infinite beam on elastic foundation be expressed as follows

7 $$ G = \frac{{E_{{\text{s}}} H_{{\text{t}}} }}{{6\left( {1 + v_{{\text{s}}} } \right)}}\psi_{{\text{t}}} $$

where $\psi_{{\text{t}}} = \frac{3}{{2\gamma_{{\text{p}}} H_{{\text{t}}} }}\frac{{\sinh \left( {\gamma_{{\text{p}}} H_{{\text{t}}} } \right)\cosh \left( {\gamma_{{\text{p}}} H_{{\text{t}}} } \right) - \gamma_{{\text{p}}} H_{{\text{t}}} }}{{\sinh^{2} \left( {\gamma_{{\text{p}}} H_{{\text{t}}} } \right)}}$; *H*_t_ = thickness of foundation shear layer; *γ*_p_ = empirical parameter. In the calculation, generally take *H*_t_ = 10*D*_P_ and *γ*_p_ = 0.7 m^-1^.

Substituting Eqs. (1) and (5)−(7) into Eq. (4) will get

8 $$ \frac{{{\text{d}}^{4} w(x)}}{{{\text{d}}x^{4} }} - \frac{{GD_{{\text{P}}} }}{{E_{{\text{p}}} I_{{\text{p}}} }}\frac{{{\text{d}}^{2} w(x)}}{{{\text{d}}x^{2} }} + \frac{{kD_{{\text{P}}} }}{{E_{{\text{p}}} I_{{\text{p}}} }}w = q(x)\frac{{D_{{\text{P}}} }}{{E_{{\text{p}}} I_{{\text{p}}} }} $$

Take any point *ξ* [*ξ* ∈ (− ∞, + ∞)] on the axis of the pipeline, the length of the micro-segment at *ξ* is d*ξ*, then the additional load acting on this segment is *q*(*ξ*)d*ξ*, and the vertical deflection *w*_0_ at any point *x* on the axis of the pipeline caused by the micro-segment load *q*(*ξ*)d*ξ* is^34^

9 $$ w_{0} = \mathop \smallint \nolimits_{ - \infty }^{ + \infty } {\text{d}}w(x) $$

where $${\text{d}}w(x) = \frac{{mq(\xi ){\text{d}}(\xi )}}{{4E_{{\text{p}}} I_{{\text{p}}} \alpha \beta \left( {\alpha^{2} + \beta^{2} } \right)}}e^{ - \alpha |x - \xi |}$$, $$m = (\beta \cos \beta |x - \xi | + \alpha \sin \beta |x - \xi |)$$, $$\alpha = \sqrt {\sqrt {\frac{{KD_{{\text{P}}} }}{{4E_{{\text{p}}} I_{{\text{p}}} }}} + \frac{{GD_{{\text{P}}} }}{{4E_{{\text{p}}} I_{{\text{p}}} }}}$$, $$\beta = \sqrt {\sqrt {\frac{{KD_{{\text{P}}} }}{{4E_{{\text{p}}} I_{{\text{p}}} }}} - \frac{{GD_{{\text{P}}} }}{{4E_{{\text{p}}} I_{{\text{p}}} }}}$$.

Further, the derivative of Eq. (9) is used to calculate the rotation angle *θ*_0_(*x*), the bending moment *M*_0_(*x*) and the shear force *Q*_0_(*x*) at any point *x* of the infinite beam.

**Figure 4:**
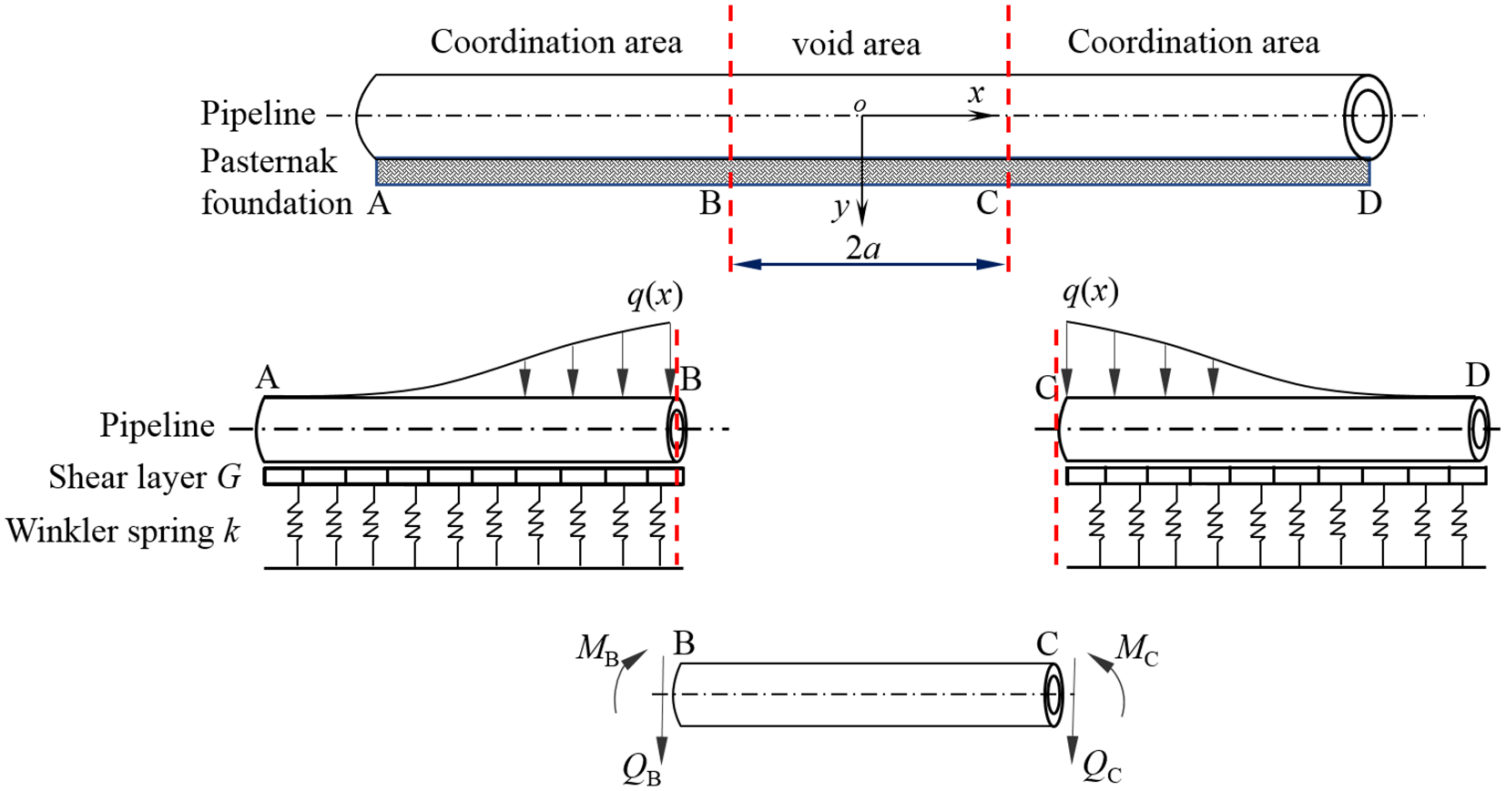
Analysis model of pipe deformation in coordination area and void area.

However, the assumption of the infinite beam model is that there is soil support under any area of the pipeline. Actually, the void may occur beneath the pipeline during the generation of tunnel volume losses, so the the above solution does not conform to the assumption of the model, and the load difference caused by the void between the pipeline and the soil should be considered in the calculation. With respect to the situation of the void between pipe and soil shown in Fig. 1, this study adopts the following ideas for analysis: assume that the width of the cavity area in Fig. 4 is 2*a*, and divide the pipeline-soil interaction into void area (i.e. BC) and coordination area (AB or CD), and regard the coordination area as a semi-infinite beam of Pasternak elastic foundation and the void area as a double-ended fixed beam, which meets the continuous conditions of internal force and displacement at the interface of the two areas.

#### Calculation of pipeline deformation in coordination area

As can be seen from Fig. 4, taking the segment CD of the coordination area on the right part of the pipeline as the analysis object, whose response is affected by the distributed load *q*(*x*) caused by ground settlement, the concentrated load *Q*_C_ and the concentrated moment *M*_C_ of the point C. Under the assumption of small deformation, the superposition method can be used to calculate the internal force and deformation of the segment CD on the right side of the pipeline. According to the superposition principle, the vertical deflection *w*(*x*) of the pipeline in the coordination area is

10 $$ w(x) = w_{1} (x) + w_{2} (x) + w_{3} (x) $$

where *w*_1_(*x*), *w*_2_(*x*) and *w*_3_(*x*) = vertical deformation of semi-infinite beam caused by *Q*_C_ and *Mc* and *q*(*x*), respectively. Notice that in the following calculation process, it is assumed that the bending moment is positive with the lower part of the beam is tensioned, the shear force is positive in the clockwise direction, and the deflection is positive in the vertical downward direction.

When calculating *w*_1_(*x*) and *w*_2_(*x*), the general solution of the vertical displacement differential equation [i.e., Eq. (8)] of the pipeline axis is obtained first. Let *q*(*x*) = 0, then there is

11 $$ \frac{{{\text{d}}^{4} w(x)}}{{{\text{d}}x^{4} }} - \frac{{GD_{{\text{P}}} }}{{E_{{\text{p}}} I_{{\text{p}}} }}\frac{{{\text{d}}^{2} w(x)}}{{{\text{d}}x^{2} }} + \frac{{kD_{{\text{P}}} }}{{E_{{\text{p}}} I_{{\text{p}}} }}w(x) = 0 $$

Integrating Eq. (11), then

12 $$ w(x) = e^{\alpha x} \left( {A_{1} \cos \beta x + A_{2} \sin \beta x} \right) + e^{ - \alpha x} \left( {B_{1} \cos \beta x + B_{2} \sin \beta x} \right) $$

In Eq. (12), if *x* tends to + ∞, then *w* = 0; and *w*(*x*) can be reduced to

13 $$ w(x) = e^{ - \alpha x} \left( {B_{1} \cos \beta x + B_{2} \sin \beta x} \right) $$

For a semi-infinite beam with concentrated load *Q*_C_ at the point C, the boundary condition expressed by the internal force at *x* = *a* is

14 $$ \left\{ {\begin{array}{*{20}l} {M_{{\left| {x = a} \right.}} = - E_{{\text{p}}} I_{{\text{p}}} \frac{{{\text{d}}^{2} w(x)}}{{{\text{d}}x^{2} }} = 0} \hfill \\ {Q_{{\left| {x = a} \right.}} = - E_{{\text{p}}} I_{{\text{p}}} \frac{{{\text{d}}^{3} w(x)}}{{{\text{d}}x^{3} }} = - Q_{{\text{c}}} } \hfill \\ \end{array} } \right. $$

Substituting Eq. (14) into Eq. (13), *w*_1_(*x*) can be obtained as

15 $$\begin{aligned} w_{1} (x) & = \frac{{Q_{{\text{C}}} D_{{\text{P}}} }}{{E_{{\text{p}}} I_{{\text{p}}} \left( {\alpha^{4} \beta + 2\alpha^{2} \beta^{3} + \beta^{5} } \right)}}e^{{ - \alpha \left( {x - a} \right)}} \\ & \quad \times \left[ {2\alpha \beta \cos \beta \left( {x - a} \right) + \left( {\alpha^{2} - \beta^{2} } \right)\sin \beta \left( {x - a} \right)} \right] \end{aligned}$$

By deriving Eq. (15), the rotation angle *θ*_1_(*x*), the bending moment *M*_1_(*x*) and the shear force *Q*_1_(*x*) due to the concentrated load *Q*_C_ at any point *x* of the semi-infinite beam can be calculated.

Additionally, for a semi-infinite beam with a concentrated moment *M*_C_ at the point C, the internal force boundary condition at *x* = *a* is

16 $$ \left\{ {\begin{array}{*{20}l} {M_{{\left| {x = a} \right.}} = - E_{{\text{p}}} I_{{\text{p}}} \frac{{{\text{d}}^{2} w(x)}}{{{\text{d}}x^{2} }} = M_{{\text{c}}} } \hfill \\ {Q_{{\left| {x = a} \right.}} = - E_{{\text{p}}} I_{{\text{p}}} \frac{{{\text{d}}^{3} w(x)}}{{{\text{d}}x^{3} }} = 0} \hfill \\ \end{array} } \right. $$

Substituting Eq. (16) into Eq. (13), *w*_2_(*x*) can be obtained as,

17 $$\begin{aligned} w_{2} (x) &= \frac{{M_{{\text{c}}} }}{{E_{{\text{p}}} I_{{\text{p}}} \beta \left( {\alpha^{4} + 3\alpha^{2} \beta^{2} - \alpha^{2} \beta + \beta^{3} } \right)}}e^{{ - \alpha \left( {x - a} \right)}} \\ & \quad \times \left[ { - \beta \left( {3\alpha^{2} - \beta } \right)\cos \beta \left( {x - a} \right) + \alpha \left( {3\beta^{2} - \alpha } \right)\sin \beta \left( {x - a} \right)} \right] \end{aligned}$$

Similarly, by deriving Eq. (15), the rotation angle *θ*_2_(*x*), the bending moment *M*_2_(*x*) and the shear force *Q*_3_(*x*) due to the concentrated moment *M*_C_ at any point *x* of the semi-infinite beam can be calculated.

**Figure 5:**
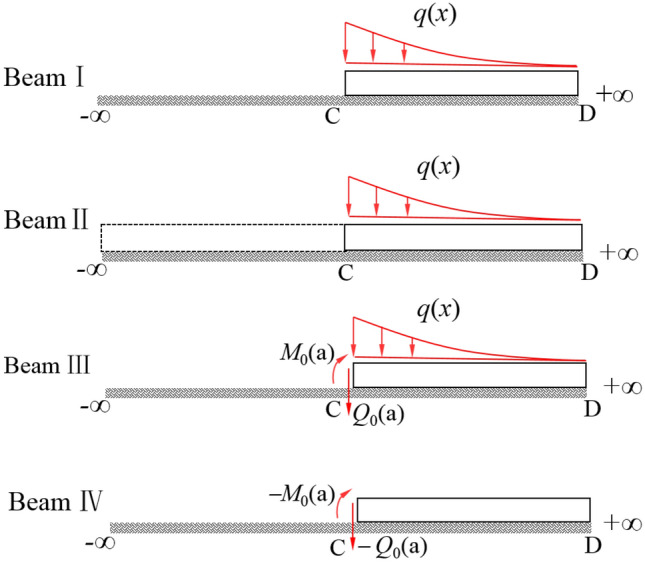
Calculation of displacement and internal force of semi-infinite beam.

The solution process of vertical displacement *w*(*x*) of beam I caused by distributed load *q*(*x*) is shown in Fig. 5, the specific steps are as follows^35^: (1) extend the left end of beam I affected by *q*(*x*) into an infinite beam II, and calculate the deflection *w*_0_(*x*) within the full length of beam II at the point *x*, and the rotation angle *θ*_0_(*a*), the shear force *Q*_0_(*a*) and the bending moment *M*_0_(*a*) at the point C (*x* = *a*) according to Eq. (9); (2) cut the beam II along point C, and take the right part of it to get the beam III, and apply the concentrated force − *Q*_0_(*a*) and the concentrated moment − *M*_0_(*a*) on beam III, and calculate the deflection increment of beam III caused by − *Q*_0_(*a*) and − *M*_0_(*a*) according to Eqs. (15) and (17); (3) due to the calculation results of beam I and beam IV are equivalent, the deflection *w*_3_(*x*) of beam IV can be calculated according to the superposition method.

#### Determination of pipeline deformation in void area

**Figure 6:**
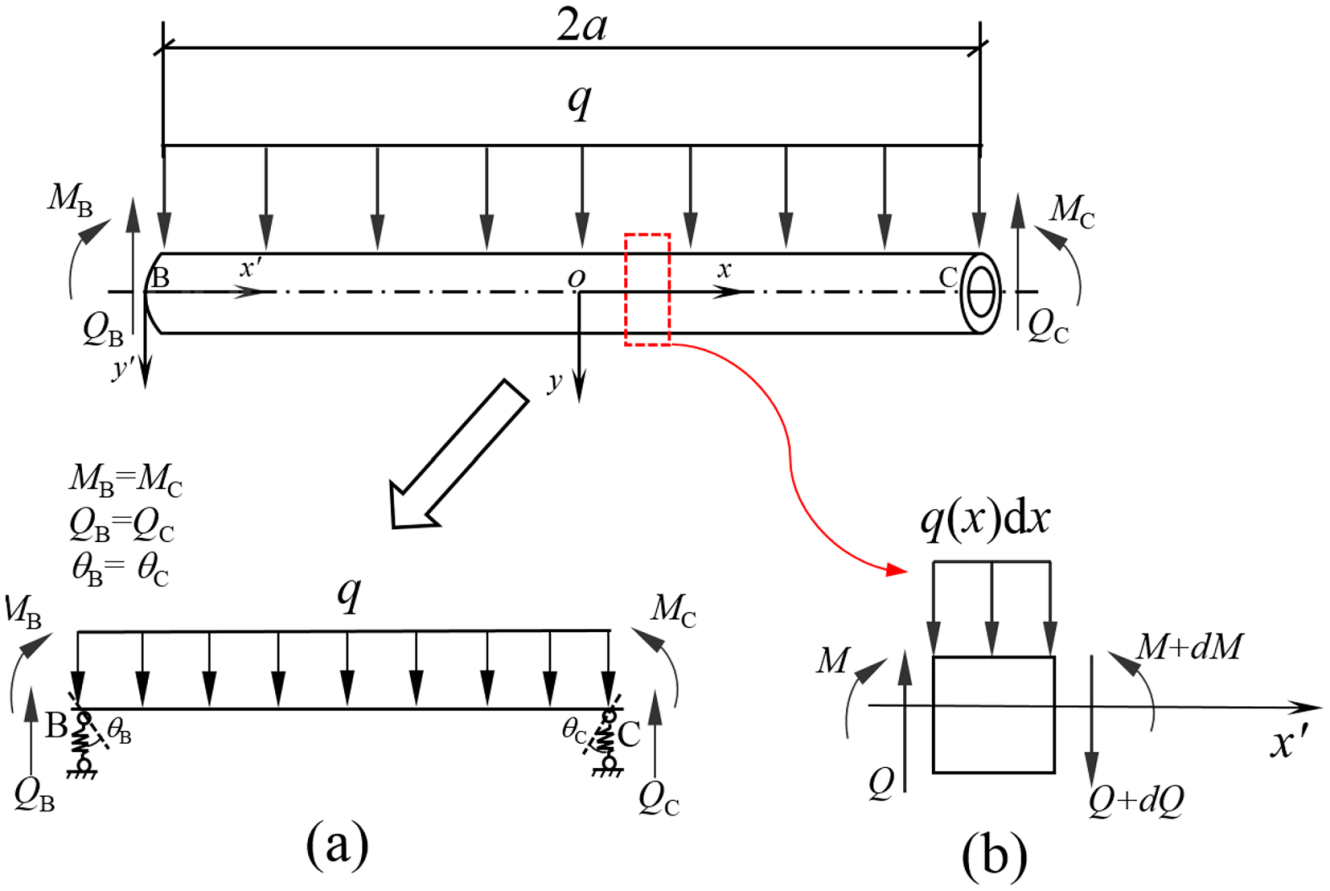
Calculation model of void zone, (**a**) simplified model; (**b**) infinitesimal element analysis.

In Fig. 4, the points B and C are the boundary points of the void area and the coordinated area of the pipeline, and the width of the void area BC is 2*a*. As the void area of pipeline is separated from the surrounding soil during the deformation process, that is, the pipeline segment loses the support of the soil, and the foundation reaction becomes zero, the load-structure model of the void area as shown in Fig. 6 can be constructed. For the convenience of calculation, it is assumed that the initial stress of the overlying soil in the void area of the pipeline is approximately the self-weight stress of the soil *γ*_s_*z*, then the dead weight load of soil acting on the pipeline is *q* = *γ*_s_*zD*_p_, where *γ*_s_ = average unit weight of soil overlying the pipeline.

For the pipeline void area in Fig. 6, if the intersection B or C of the elastic foundation and the coordination area is replaced by a fixed support, the void area of the pipeline transmits shear force and bending moment to the coordination area through this support. Moreover, due to the rotation during the deformation of the pipeline, it is assumed that the fixed support has an initial rotation angle *θ*_C_ (or *θ*_B_) during the calculation process. In order to facilitate formula derivation, a new coordinate system *x'* − *y'* is established, with point B on the pipeline axis as the coordinate origin, and the transformation relationship between the new and old coordinate systems is *x'* = *x* + *a*, *y'* = *y*.

According to the structural mechanics, the vertical deflection of the pipeline void area BC at any point *x'* is^36^

18 $$ w\left( {x^{\prime}} \right) = \frac{1}{{E_{{\text{p}}} I_{{\text{p}}} }} - \left( {\frac{q}{24}x^{{\prime}{4}} - \frac{qa}{6}x^{{\prime}{3}} + \frac{{qa^{2} }}{6}x^{{\prime}{2}} } \right) + \frac{{\theta_{{\text{C}}} }}{2a}x^{{\prime}{2}} - \theta_{{\text{c}}} x^{\prime} + S_{c} $$

The bending moment at the point *x'* = 2*a* is calculated by deriving Eq. (18),

19 $$ M_{{\left| {x^{\prime} = 2a} \right.}} = \frac{{ - \theta_{{\text{C}}} E_{{\text{p}}} I_{{\text{p}}} - \frac{1}{3}qa^{3} }}{a} $$

where *S*_c_ = vertical displacement of soil at point C in the coordination area. The deformation of the pipeline and the soil at point C is always consistent, namely,

20 $$ S_{{\text{C}}} = w_{{\text{C}}} $$

where *w*_C_ = vertical deflection at point C of the pipeline in the void area.

### Determination of the range of pipeline-soil void area

**Figure 7:**
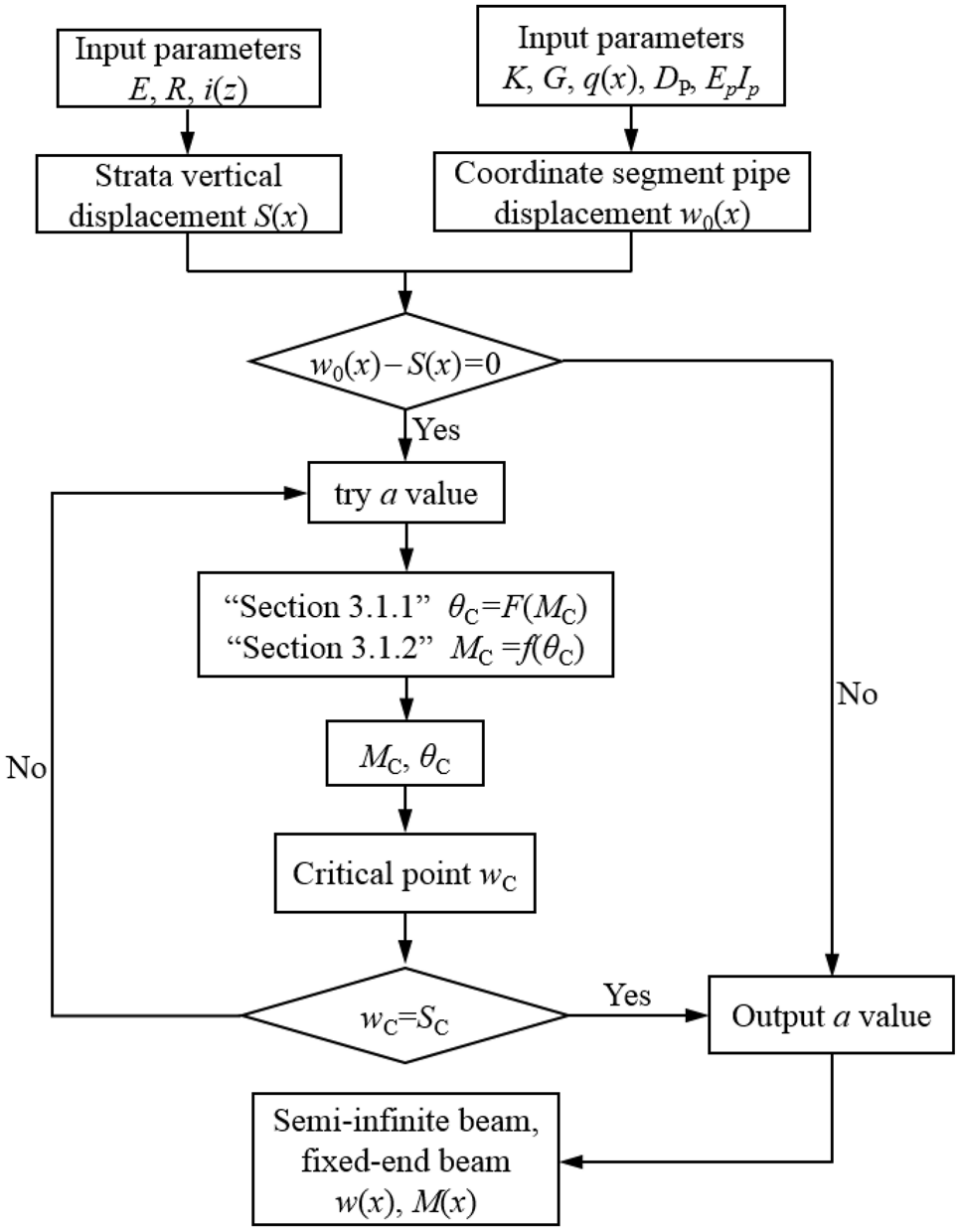
Flow chart for calculation of the range of pipeline-soil void area.

According to the flowchart shown in Fig. 7, the following trial calculation method can be used to determine the width 2*a* of void area: (1) by combining Eqs. (3) and (9), the vertical displacement *w*_0_(*x*) at the axis of the pipeline and the stratum settlement *S*(*x*) at the buried depth of the pipeline are calculated respectively, and solve the difference *S*_rel_(*x*) = *w*_0_(*x*)* − S*(*x*); (2) judge whether there is an *a*_0_ to satisfy *S*_rel_(*a*_0_) = 0 based on the iterative method, if *a*_0_ does not exist, the deformation between the full length of the pipe and the soil is considered to be coordinated, and the deformation result of the infinite beam can be directly output as the deformation of the pipeline, i.e. *w*(*x*) = *w*_0_(*x*); conversely, if there is such a *a*_0_, the pipeline-soil detachment is considered to occur at a certain critical point C (or B), and try to take *a* = *a*_0_; from the contents of “Calculation of pipeline deformation in coordination area” and “Determination of pipeline deformation in void area”, it can been seen that the rotation angle *θ*_C_ at point C (or B) of the void area of the pipeline is a function of the bending moment *M*_C_, that is, *θ*_C_ = *F*(*M*_C_), and the bending moment *M*_C_ of the coordination area of the pipeline is a function of the rotation angle *θ*_C_, that is, *M*_C_ = *f*(*θ*_C_); (3) by combining the two functions in step (2), *M*_C_ and *θ*_C_ can be solved, and then the vertical deflection *w*_C_ at point C (or B) can be obtained from Eq. (18); (4) determine whether *w*_C_ is consistent with the vertical displacement *S*_C_ of the stratum, if it is consistent, output *a*; if not, then try to take *a* again, repeat steps (1)–(3) until the error between *w*_C_ and *S*_C_ satisfies |*w*_c_-*S*_c_|/|*S*_c_|< 0.25%.

In practical engineering, when calculating the range of pipeline-soil void zone, the free deformation *S*(*x*) of soil in the buried depth of pipeline is determined by monitoring data, and the stratum displacement load is calculated by *S*(*x*), and the initial pipeline deformation is calculated by infinite elastic foundation beam method. Then the void area is determined by the pipeline-soil separation boundary condition *w*(*x*) = *S*(*x*), and the overlying load on the pipeline is changed, and the pipeline deformation is solved by the method of semi-infinite beam and fixed support beam, and the unique range of pipeline-soil void area can be obtained by repeated iteration.

## Verification by comparison

### Centrifuge model test

To verify the proposed analytical method, the deformation and bending moment of the pipeline predicted by this method and other analytical methods are compared with the experimental results of the centrifuge model test of the tunnel excavation beneath the existing pipeline in sandy stratum. The centrifugal acceleration of the test is 60 g, and the other dimensions and characteristics of the model are as follows: *D*_p_ = 31.75 × 10^−3^ m, *L* = 1.15 m, *E*_p_ = 69GPa, *I*_p_ = 2.1439 × 10^-8^m^4^, *z* = 0.096 m, *R* = 0.05 m, *z*_0_ = 0.25 m, *γ*_s_ = 15.4kN/m^3^, *E*_*s*_ = 10 MPa, *v*_*s*_ = 0.3. It should be noted that in order to avoid systematic errors in the calculation results of the pipeline caused by the empirical selection of the stratigraphic parameters, the stratigraphic deformation *S*(*x*) is directly selected from the fitted curve obtained from the measured values of the free settlement of the strata at the location of the pipeline axis depth in the literature^8^, where *S*_max_(*z*) = 0.84 × 10^−3^ m, *i*(*z*) = 0.1225 m*.*

**Figure 8:**
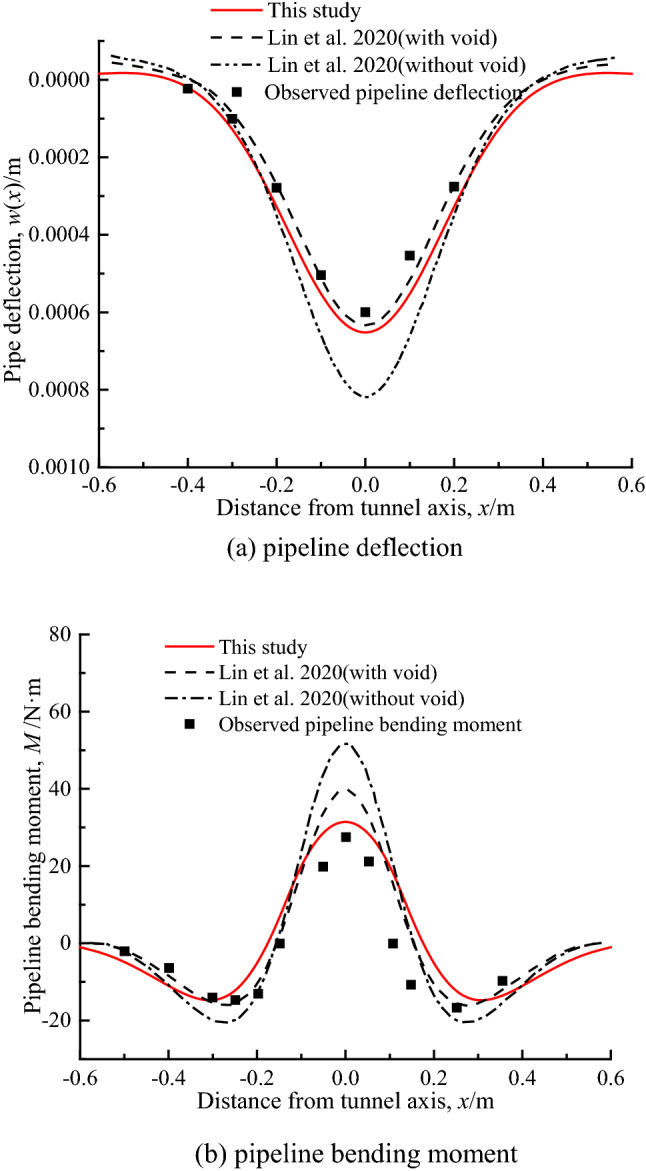
Comparison of calculation results of pipeline deflection and bending moment.

Figure 8 shows the comparison of the results of the pipeline responses (in terms of deflection and bending moment) for the analytical method of this study and Lin et al.^26^ as well as the experimental test, and also gives the calculation results of Lin et al.^26^ without considering the detachment of pipeline and soil. It can be seen that the calculated values in this study is in good agreement with the experimental values and the estimated values of Lin et al. The maximum deflections of the pipeline is slightly larger than the calculated values of Lin et al., but the maximum bending moment of the pipeline is closer to the experimental value. The comparison results can verify the reliability of the method in this study.

### Laboratory model test

Wang et al.^37^ conducted the large-scale model tests to simulate the effects of vertical underpass tunnel construction in sandy soils on the deformation of existing pipelines. The dimensions and characteristics of the model are as follows: *D*_p_ = 0.2 m, *L* = 2 m, *E*_p_ = 2.3GPa, *I*_p_ = 4.637 × 10^-5^m^4^, *z* = 0.75 m, *γ*_s_ = 14.7kN/m^3^, *E*_s_ = 2.5 MPa, *v*_s_ = 0.3, *S*_max_(*z*) = 8.795 × 10^−3^ m, *i*(*z*) = 0.2993 m. In this test, the movement of the sliding plate at the bottom of the model box was used to simulate the Gaussian settlement curve caused by tunnel excavation, and the settlement rod was set to measure the deformation of the pipeline and the free deformation of the stratum around the depth of the pipeline axis.

**Figure 9:**
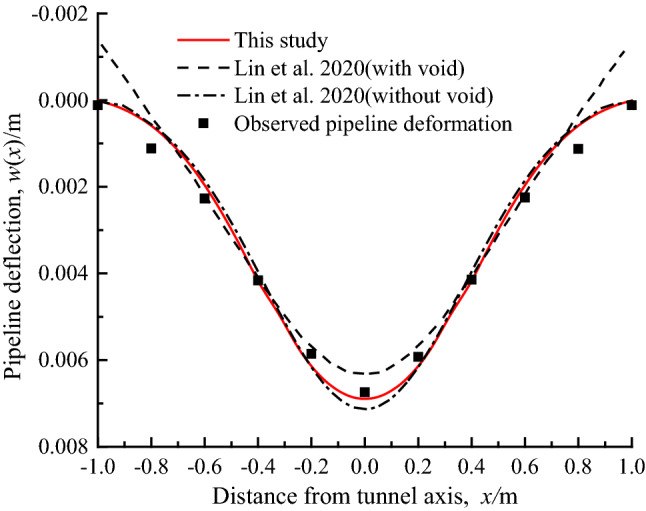
Comparison of calculation results of pipeline deflection.

Figure 9 shows the comparison of the results of the pipeline deformation for the analytical method of this study and Lin et al.^26^, and also provides the calculated results of Lin et al.^26^ without void area. It can be seen that the trend of measured pipeline deflection is relatively consistent with the calculated value. However, for the pipeline segment *x* ≥ 4*D*_P_, there are certain differences between the experimental data and the calculated value of Lin et al.^26^, but the calculated results of the proposed method are more consistent with the experimental results. Additionally, from the test results, a void area with half width of the settlement through of about 0.33 m is formed beneath the pipeline, which is consistent with *a* = 0.32 m calculated by the proposed method, which further verifies the rationality of the theoretical assumption and derivation process in this study.

### Field test

Jia et al.^9^ conducted settlement observation on the construction process of the underpass cable pipeline in the first phase of Shenzhen Metro project, and the test pipeline was located in the sandy clay. In this study, the monitoring results of pipeline deformation after the excavation of the left line tunnel are adopted for verification. The dimensions and characteristics of the model are as follows: *D*_p_ = 3 m, *L* = 100 m, *E*_p_ = 25GPa, *I*_p_ = 1.7329m^4^, *z* = 8.7 m, *R* = 3 m, *z*_0_ = 14.4 m, *γ*_s_ = 15.57kN/m^3^, *E*_s_ = 8.2 MPa, *v*_s_ = 0.3, *S*_max_(*z*) = 0.012185 × 10^−3^ m, *i*(*z*) = 6.48 m.

**Figure 10:**
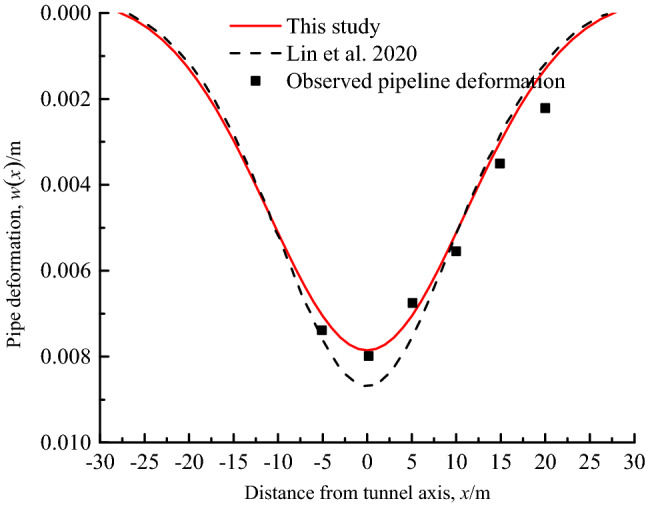
Comparison of calculation results of pipeline deflection.

Figure 10 shows the comparison between the calculation results from the proposed method and Lin et al.^26^ and the field test data. Through the calculation, it is found that there is no void area at the bottom of the pipeline, which is due to the larger buried depth of the pipeline and the larger displacement load of the overlying soil relative to the stratum, which makes the central deformation of the pipeline larger and is easy to contact with the soil beneath the pipeline. As can be seen from Fig. 10, the calculation results in this study is in good agreement with the observed value, and their deviation is smaller than that of the calculation method from Lin et al.^26^, so it can be seen that this method is also suitable for sandy clay.

## Parametric investigation

In order to discuss the influence of the changes of main physical parameters on the deformation of the pipeline considering the void effect, the following examples are used for comparative analysis. Tunnel calculation parameters: *z*_0=_15 m, *R* = 3 m, *ε* = 3%; pipeline calculation parameters: *D*_P_ = 1.9 m, *z* = 4.8 m, *E*_p_ = 69GPa, *I*_p_ = 3.19 × 10^-1^m^4^; soil calculation parameters: *E*_s_ = 10 MPa, *v*_s_ = 0.3, *γ*_s_ = 15.4kN/m^3^.

### Pipeline bending stiffness ***E***_p_***I***_p_

**Figure 11:**
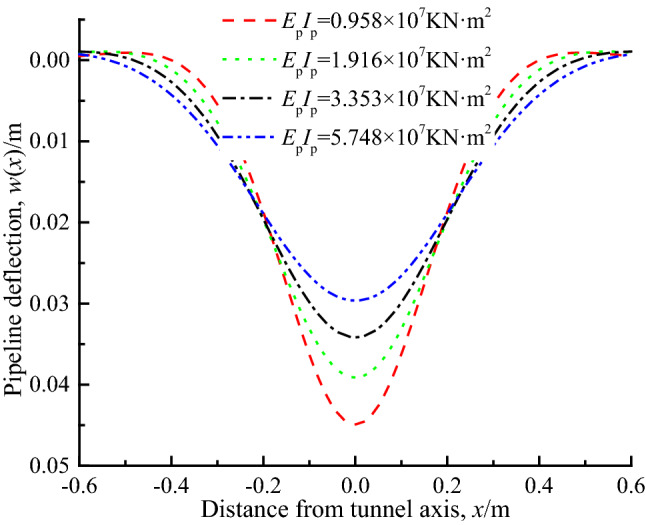
Pipe deflection under different pipeline stiffness.

Figure 11 shows the pipeline deflection curve when pipeline bending stiffness *E*_p_*I*_p_ is 0.958 × 10^7^ KN m^2^, 1.916 × 10^7^ KN m^2^, 3.353 × 10^7^ KN m^2^ and 5.748 × 10^7^ KN m^2^. It can be seen that as the bending stiffness of the pipeline increases, the pipeline deflection gradually decreases, and the proportion of reduction gradually decreases. The reason is that the bending stiffness of the pipeline directly affects its ability to bear the ground load and resist bending deformation, that is to say, the greater its stiffness, the stronger its ability to share the load, so that the pipeline can deform less in the process of pipeline-soil interaction.

### Soil elastic modulus ***E***_s_

**Figure 12:**
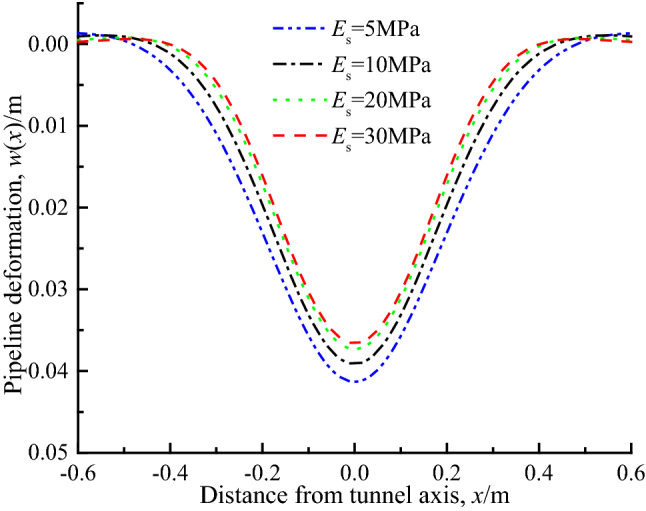
Pipe deflection under different soil modulus.

Figure 12 shows the deflection curve of the pipeline with the elastic modulus of soil *E*_s_ of 5 MPa, 10 MPa, 20 MPa and 30 MPa respectively. It can be seen that with the increase of elastic modulus, the deflection of pipeline decreases gradually, but the decrease rate is less than that of pipeline bending stiffness (by comparing Figs. 10 and 11). Obviously, the elastic modulus of soil *E*_*s*_ affects the pipeline deflection mainly by changing the foundation parameters *k* and *G*. From the parameters *k* and *G* in Eqs. (6) and (7), it is known that the parameters *k* and *G* increase with the increase of elastic modulus, so as to enhance the ability of soil to resist deformation. Additionally, it can be seen from Eq. (8) that the increase of *k* and *G* results in the gradual decrease of the load *q*(*x*) acting on the pipeline caused by the formation settlement, which is also the reason for the reduction of the pipeline deflection.

### Pipeline-tunnel spacing ratio ***Z***_T_/***D***_P_

In order to simplify the analysis, the normalized parameter *Z*_T_/*D*_P_ is used to represent the pipeline-tunnel spacing ratio, wherein *Z*_T_ = distance from pipeline axis to tunnel axis.

**Figure 13:**
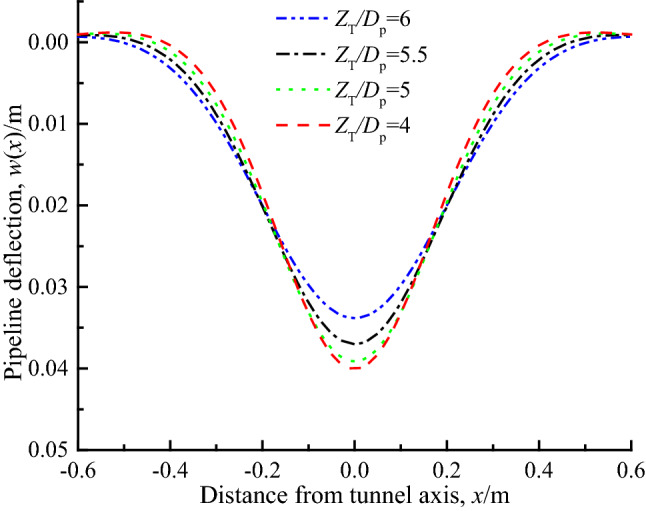
Pipe deflection under different pipe-tunnel spacing.

Figure 13 shows the pipeline deflection curves with *Z*_T_/*D*_P_ of 4, 5, 5.5 and 6 respectively. It can be seen that with the increase of the pipeline-tunnel spacing ratio, the maximum deflection of the pipeline decreases, and its reduction rate gradually increases. The main reason is that when the distance between pipeline and tunnel increases, the pipeline are less affected by tunnel excavation, and the stratum deformation at the depth of the pipeline axis caused by the loss of tunnel excavation also decreases, which in turn causes the formation deformation load borne by the pipeline is correspondingly reduced, thereby reducing the pipeline deflection.

### Formation loss rate *ε*

**Figure 14:**
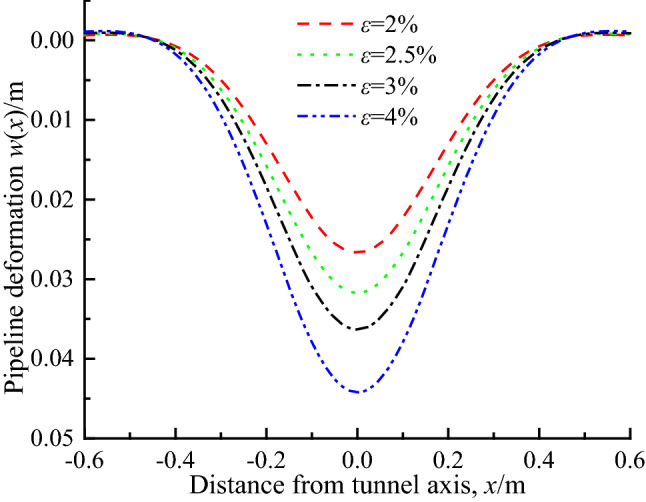
Pipe deflection under different soil loss.

Figure 14 shows the pipeline deflection curve with the formation loss rate *ε* of 2%, 2.5%, 3% and 4% respectively. It can be seen that the influence of formation loss on pipeline deflection is more significant than the above three factors. The reason is that the formation loss rate is directly related to the ground settlement. With the increase of formation loss rate, the deflection of pipeline axis increases obviously. For example, when *ε* increases from 2 to 4%, the deflection of pipeline axis increases by 65.6%. It can be explained that the formation loss rate *ε* affects the pipeline deflection by changing the free subsidence of strata, which shows that the larger *ε* is, the greater the ground settlement at the axis of the pipeline is, so the greater the stratum load acting on the pipeline is, and the greater the pipeline deformation is. This phenomenon is consistent with the conclusion of Franza et al.^38^ based on the two-stage analysis method of continuum that “the volume loss of tunnel will be accompanied by the formation of void gap, and may lead to the nonlinear trend of structural deformation”.

## Discussion on the range of the void area

As can be seen from the aforementioned verification section, the maximum difference in the calculated pipeline deflection between the two cases of considering the pipeline-soil void and not considering the pipeline-soil void is 20%, so it is important to consider the pipeline-soil voiding phenomenon to calculate the pipeline deformation more accurately. Moreover, the range of void area will be affected by material parameters such as pipeline stiffness, elastic modulus of soil and geometric parameters such as pipeline-tunnel spacing. For this reason, this study will further compare and analyze the factors that affect the scope of the void area.

To compare the extent of detachment between pipeline and soil in the void area more intuitively, the normalization parameter is defined, i.e., *R*_r,max=_(*S*_max_* − w*_max_)_/_*S*_max_, wherein *S*_max_, *w*_max_ are the free subsidence of the stratum in the buried depth of the pipeline and the maximum pipeline deflection, respectively; *R*_r,max_ is the ratio of the relative maximum displacement between the pipeline and the surrounding soil to the maximum soil displacement, indicates the relative magnitude of the pipeline-soil displacement difference.

### Pipeline bending stiffness ***E***_P_***I***_P_

**Figure 15:**
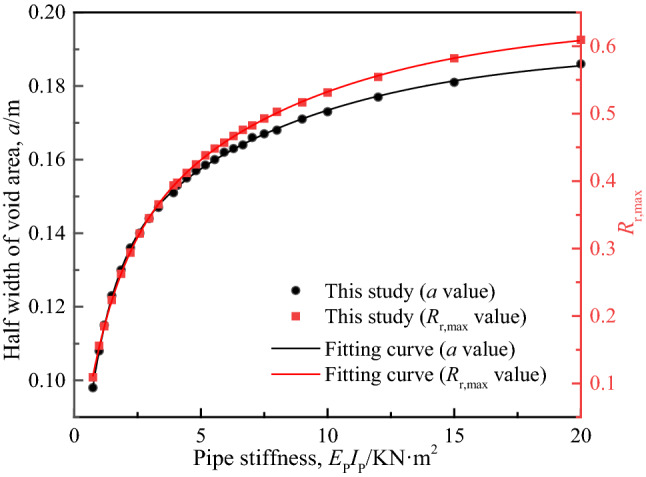
Relative detachment extent between pipeline and soil under different pipeline stiffness.

Select the calculation results of 26 groups of different pipeline bending stiffness *E*_P_*I*_P_, and draw the relationship curves of *R*_r,max_ versus *a* and *R*_r,max_ versus *E*_P_*I*_P_, as shown in Fig. 15. It can be seen that *a* and *R*_r,max_ are approximately exponentially related to *E*_P_*I*_P_, the growth of *a* increases gradually with the increase of *E*_P_*I*_P_ but becomes gentle, and *R*_r,max_ tends to grow steadily. This shows that the extent of pipeline-soil detachment increases with the increase of pipeline stiffness, but when the stiffness increases to a certain amount, its impact on the void section gradually decreases. The reason is that when the pipeline EpIp increases, the relative stiffness of the pipeline and the soil increases, making it easier for uncoordinated deformation to occur between the pipeline and the soil. Meanwhile, due to the pipeline in the void area is only considered to be subject to the self-weight of the overlying soil layer, the increase of the pipeline stiffness makes the pipeline deflections in the void area decrease, while the relative displacement ratio *R*_r,max_ at the midpoint of the void area of pipeline increases significantly, that is to say, the extent of pipeline-soil detachment increases.

### Soil elastic modulus ***E***_s_

**Figure 16:**
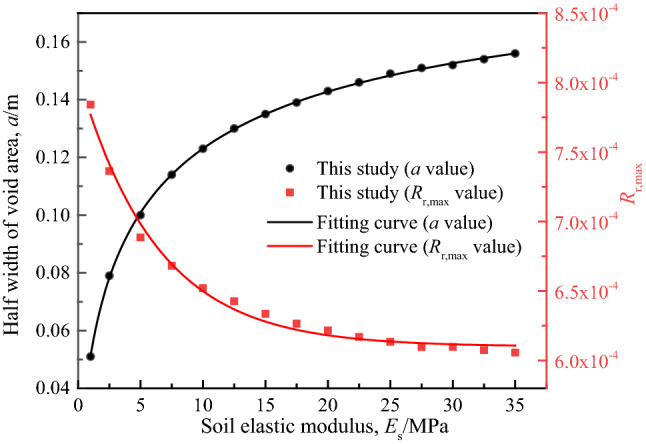
Relative detachment extent between pipeline and soil under different soil elastic modulus.

Select the calculation results 15 groups of different soil elastic modulus *E*_s_, and draw the relationship curves of *R*_r,max_ versus *a* and *R*_r,max_ versus *E*_s_, as shown in Fig. 16. It can be seen that *a* increases with the increase of *E*_s_, and the increasing trend gradually tends to be gentle from rapid, but *R*_r,max_ are exponentially negatively correlated with *E*_s_, until *E*_s_ increases to a certain amount, *R*_r,max_ tend to be stable. This shows that increasing the elastic modulus of soil may cause an appropriate increase in the void width, but the extent of detachment between pipeline and soil is reduced, which is conducive to the safety of the pipeline. Obviously, increasing the elastic modulus of the soil is equivalent to reducing the stiffness difference between pipeline and soil, which is conducive to the coordinated deformation of the two.

### Pipeline-tunnel spacing ratio ***Z***_T_/***D***_P_

**Figure 17:**
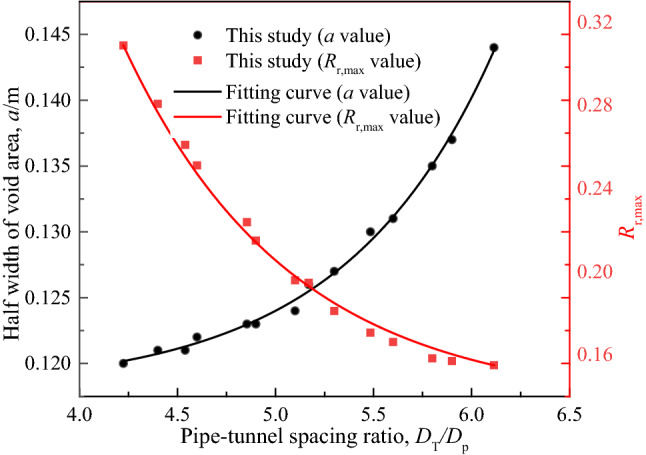
Relative detachment extent between pipeline and soil under different pipe-tunnel spacing ratios.

Select the calculation results of 14 groups of different pipeline-tunnel spacing ratios *Z*_T_/*D*_P_, and draw the relationship curves of *R*_r,max_ versus *a* and *R*_r,max_ versus *Z*_T_/*D*_P_, as shown in Fig. 17. It can be seen that *a* increases with the increase of *Z*_T_/*D*_P_, and the increasing trend is gradually obvious, which is caused by the decrease of the width *i*(*z*) of the settlement trough, but *R*_r,max_ is the opposite trend. That is, when the pipeline is close to the tunnel axis, the maximum deformation of the stratum increases significantly, and the deformation of the center of the pipeline is much less than the deformation of the stratum, which shows that the detachment extent of pipeline and soil is high, and the impact on the safety of the pipeline is intensified.

## Limitations

This study assumes that the pipeline is not affected by lateral earth pressure, and only considers that it is affected by the ground displacement load and the gravity of the overlying soil, and in the actual project, the pipeline will also be affected by the construction caused by tunnel excavation and the structure of the soil. The mechanism of pipe-soil interaction is more complex, and it is not simply to apply displacement load on the pipeline. In addition, the Peck empirical formula is used to calculate the stratum displacement in this study, it is assumed that the foundation soil is a homogeneous isotropic linear elastic body in semi-infinite space, but in practical engineering, the foundation soil is anisotropic, stratified and elastic–plastic. Furthermore, the formation displacement has three-dimensional characteristics, which should include both vertical and horizontal displacement, but only the vertical displacement is considered in this study.

## Conclusions

In this study, an analytical method for calculating the deflection of overlying pipeline caused by tunnel excavation based on the semi-infinite elastic foundation beam theory is proposed, and the main factors affecting the deformation of pipelines and the range of the void area are discussed. The main conclusions are as follows:

1. By considering the pipeline-soil void, the deflection of the pipeline is reduced, while the conventional calculation method that does not consider the pipeline-soil void is conservative.
2. The maximum deflection of pipeline increases with the increase of formation loss rate, and decreases with the increase of the pipeline-tunnel spacing, the pipeline bending stiffness and the soil elastic modulus, but their decreasing rate slows down with the increase of these parameters;
3. The range of void area increases with the increase of the pipeline bending stiffness, the soil elastic modulus and the pipeline-tunnel spacing, and the extent of pipeline-soil detachment increases with the increase of the pipeline bending stiffness, but decreases with the increase of the soil elastic modulus and the pipeline-tunnel spacing.

## Data Availability

The data used to support the findings of this study are available from the corresponding author upon reasonable request.

## List of symbols

*x*: Lateral horizontal distance from the tunnel axis
*q*(*x*): Distributed load acting on the pipeline at coordinate *x*
*G*: Pasternak foundation reaction coefficient
*k*: Coefficient of subgrade reaction
*S*(*x*), *S*_0_(*x*): Ground settlement and ground surface settlement at coordinate *x*, respectively
*R*: Tunnel radius
*ε*: Formation loss rate caused by tunnel excavation
*i*_0_, *i*(*z*): Width coefficient of ground settlement trough at *z* = 0 and any depth *z*, respectively
*z*_0_: Buried depth of tunnel and pipeline, respectively
*z*: Depth from the ground surface
*n*: Influence coefficient related to tunnel radius and soil conditions
*S*_max_(*z*): Maximum ground settlement above the tunnel axis at depth *z*
*w*(*x*): Vertical deflection of pipeline at coordinate *x*
*w*_1_(*x*), *w*_2_(*x*), *w*_3_(*x*): Vertical deflection caused by *Q*_C_, *Mc* and *q*(*x*), respectively
*D*_P_: Outer diameter of the pipeline
*E*_p_: Elastic modulus of pipeline
*I*_p_: Section moment of inertia
*G*: Pasternak foundation shear stiffness
*E*_s_, *v*_s_: Elastic modulus and Poisson's ratio of soil, respectively
*η*: Depth correction factor
*H*_t_: Thickness of foundation shear layer
*γ*_p_: Empirical parameter
*a*: Half width of the void area
*γ*_s_: Average unit weight of soil overlying the pipeline
*S*_c_: Vertical displacement of soil at point C in the coordination area
*w*_C_: Vertical deflection at point C of the pipeline in the void area
*θ*_0_(a), *Q*_0_(a) and *M*_0_(a): Rotation angle, the shear force and the bending moment at the point C, respectively
*Q*_C_, *M*_C_: The concentrated load and the concentrated moment of the point C, respectively
*Z*_T_: Distance from pipeline axis to tunnel axis
*L*: Length of pipeline
*R*_r,max_: Ratio of the relative maximum displacement between the pipeline and the surrounding soil to the maximum soil displacement
